# Association Between Sugar‐Sweetened Beverage Consumption and Frailty Among Older Adults With Hypertension: Evidence From the National Health and Nutrition Examination Survey 1999–2020

**DOI:** 10.1002/fsn3.4679

**Published:** 2024-12-30

**Authors:** Si Cao, Youjie Zeng, Sai Zhou, Wenming Song, Gong Chen

**Affiliations:** ^1^ Department of Anesthesiology, The Third Xiangya Hospital Central South University Changsha Hunan China; ^2^ Department of Academic Affairs Changsha Medical University Changsha Hunan China; ^3^ Department of Chinese and Western Medicine Combined, Changde Hospital, Xiangya School of Medicine Central South University (The First people's Hospital of Changde City) Changde Hunan China

**Keywords:** frailty, hypertension, NHANES, older adults, sugar‐sweetened beverage

## Abstract

Frailty is a condition characterized by increased vulnerability to adverse health outcomes, particularly among older adults. With the significant prevalence of hypertension and the consumption of sugar‐sweetened beverages (SSBs) in this demographic, it is essential to explore their potential combined effects on frailty. This cross‐sectional study utilized data from the National Health and Nutrition Examination Survey (NHANES) spanning 1999–2020, involving 13,465 hypertensive adults aged 60 and above. Frailty was assessed using a Frailty Index based on 49 deficits, categorizing individuals as frail if their score was ≥ 0.25. SSB consumption was measured through 24‐h dietary recalls. Multivariate logistic regression analyses, adjusted for various confounding factors including age, sex, body mass index (BMI), socioeconomic status, and lifestyle choices, indicated that SSB consumption was independently associated with an increased likelihood of frailty (odds ratio = 1.18, 95% confidence interval: 1.02–1.37, *p* = 0.02). Additionally, restricted cubic spline curve fitting revealed a linear relationship between SSB intake and frailty levels. Our findings suggested that SSB consumption was associated with frailty in older hypertensive adults, emphasizing the need for further research on the underlying mechanisms and potential interventions.

## Graphical abstract

The relationship between sugar‐sweetened beverages and frailty in elderly hypertensive individuals: Even after adjusting for potential confounders, sugar‐sweetened beverage consumption was independently associated with increased frailty risk among older adults with hypertension (OR = 1.18, 95% CI: 1.02–1.37, *p* = 0.02). A linear relationship was observed between sugar‐sweetened beverage consumption and frailty risk. The study suggests that older adults with hypertension who consume sugar‐sweetened beverages are at an increased risk of frailty.

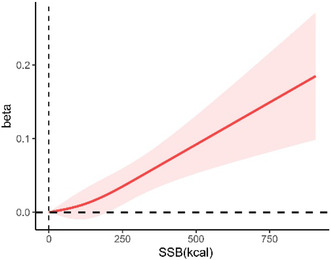

## 1 Introduction

Frailty, defined as a state of increased vulnerability to adverse health outcomes, is a growing concern among older adults worldwide (Walsh et al. 2023). This multidimensional syndrome is characterized by a decline in physiological reserves and functional capabilities, which can lead to falls, disability, hospitalization, and even mortality (Hoogendijk et al. 2019). Identifying and addressing modifiable risk factors for frailty is essential for promoting healthy aging and alleviating the burden on healthcare systems.

Recent findings from a population‐based study in Southern Italy revealed that the overall prevalence of physical frailty among older adults was 14.8%, with a notable increase in prevalence correlating with age and a higher incidence in females compared to males (Castellana et al. 2021). One such modifiable factor that has gained significant attention is the consumption of sugar‐sweetened beverages (SSB). These drinks, which include sodas, fruit drinks, and sports drinks, are major sources of added sugars in modern diets. Excessive intake of SSBs is linked to obesity, type 2 diabetes, cardiovascular disease, and certain cancers (Bleich and Vercammen 2018; Malik and Hu 2022).

Hypertension, another prevalent condition among older adults, is also a potential risk factor for frailty. Uncontrolled hypertension can result in vascular damage, organ dysfunction, and cognitive impairment, all of which contribute to the development of frailty (Canavan and O'Donnell 2022; Jung et al. 2021; Tadic, Cuspidi, and Hering 2016). Given the high prevalence of both hypertension and SSB consumption in older populations, it is crucial to explore their potential synergistic effects on frailty. Most previous studies have focused on general populations, neglecting the specific interactions between SSB consumption and hypertension in older adults.

This cross‐sectional study utilizes data from the National Health and Nutrition Examination Survey (NHANES) spanning 1999 to 2020 to investigate the association between SSB consumption and frailty in older adults with hypertension. The findings aim to inform targeted interventions and dietary recommendations for this vulnerable population.

## 2 Methods

### 2.1 Study Population

**FIGURE 1:**
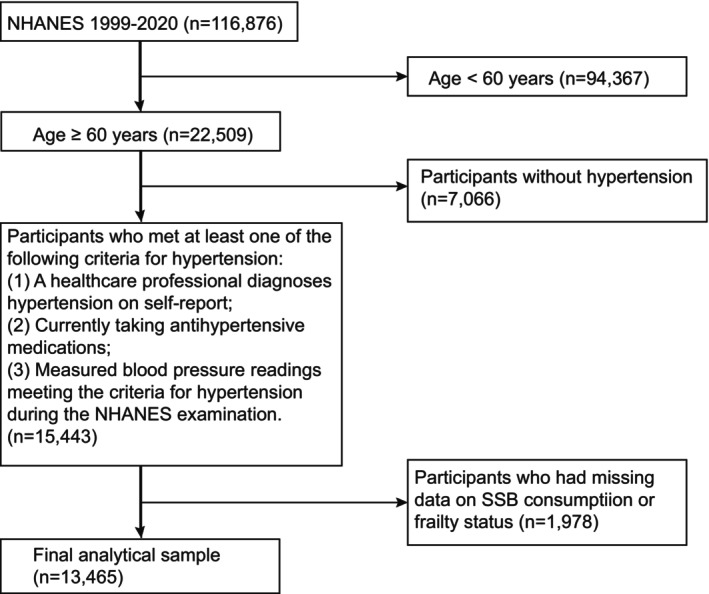
The flow chart of inclusion and exclusion criteria in the study.

This cross‐sectional study utilized data from the National Health and Nutrition Examination Survey (NHANES) spanning from 1999 to 2020. The NHANES survey collected health and nutritional information about the U.S. non‐institutionalized civilian population from the Centers for Disease Control and Prevention (CDC). The study population included older adults aged 60 years and above who met at least one of the following criteria for hypertension: (1) Self‐reported diagnosis of hypertension by a healthcare professional; (2) current use of antihypertensive medications; (3) measured blood pressure readings meeting the criteria for hypertension during the NHANES examination. Participants were excluded if they had missing data on key variables, such as SSB consumption and frailty status. Details of the study sampling and exclusion criteria are shown in Figure 1. The final analytical sample consisted of 13,465 older adults with hypertension. All participants provided written informed consent prior to the data analysis, which was approved by the National Center for Health Statistics (NCHS)' Research Ethics Review Board.

### 2.2 Frailty Index

According to previous literature (Hakeem, Bernabe, and Sabbah 2021), the Frailty Index score was calculated based on 49 deficits covering multiple systems, including cognition, dependence, depression, comorbidities, hospital use, general health, physical functioning, anthropometry, and laboratory results. These included questions about confusion and memory, difficulty performing daily activities, depressive symptoms, self‐reported health, hospitalization, health care use, prescribed medications, handgrip strength, body mass index (BMI), and various laboratory values. The Frailty Index score was determined by dividing the number of deficits present by the total number of potential deficits. Two categories were established to classify frailty status: non‐frail (Frailty Index < 0.25) and frail (Frailty Index ≥ 0.25), as defined in the previous study (Li et al. 2023).

### 2.3 SSB

Two days of 24‐h recall interviews were conducted to obtain detailed food and drink intake information. Only the first‐day recall interview in the mobile examination center (MEC) was adopted. SSB included soft drinks, fruit drinks (not 100%), sports drinks, energy drinks, nutritional drinks, smoothies, grain drinks, bottled water, carbonated water, sweetened coffee, and sweetened tea. The grams of added sugar are multiplied by 4 kcal to determine the total energy intake of SSB.

### 2.4 Covariates

Sociodemographic factors included age (as a continuous variable in years), sex, and ethnicity, categorized into Hispanic, non‐Hispanic Black, non‐Hispanic White, and other races. Education was grouped into five levels: < 9th grade, 9th–11th grade, high school graduate, college, and college graduate. The poverty‐income ratio (PIR) represented income levels and was used as a continuous variable. Three groups of smokers were categorized: never smokers (smoked < 100 cigarettes), previous smokers, and current smokers. BMI was extracted directly from the database. Finally, physical activity was expressed as daily metabolic equivalent tasks (MET).

### 2.5 Statistical Analysis

All descriptive analyses and regression modeling were done using R version 4.3.0 and the R package “Survey.” To consider the intricate survey design, nonresponse to the survey, and post‐stratification, sampling weights and design variables were utilized in all analyses to acquire unbiased estimates of national prevalence and variance. Means and standard error (SEM) were used to represent continuous quantitative variables, while numbers and percentages were used for qualitative variables. A *t*‐test and chi‐squared test were used to assess significance based on the variables (Table 1). Logistic regression was employed to evaluate the connection between covariates and frailty (Table 2). Furthermore, both unadjusted and adjusted multivariate regression models explored the independent association between SSB and frailty. In the Model I (crude model), nothing was adjusted. Age, sex, and BMI were adjusted in Model II (minimally adjusted model). In Model III (fully adjusted model), all the significant covariates in univariate analysis were adjusted (Table 3). Since SSB consumption and frailty index score were continuous variables, a curve relationship between SSB consumption and frailty index score was evaluated through restricted cubic spline (RCS) curve fitting (Figure 1). *p* < 0.05 was considered statistically significant in all analyses.

**TABLE 1:** Characteristics of study participants.

Variable	Non‐consumers of SSB (*n* = 7247)	Consumers of SSB (*n* = 6218)	*p*
Age, years [mean (SEM)]	71.26 (0.13)	69.97 (0.12)	< 0.0001
Sex [*n* (%)]			0.003
Male	3355 (41.26)	3027 (44.93)	
Female	3892 (58.74)	3191 (55.07)	
BMI, kg/m^2^ [mean (SEM)]	29.41 (0.11)	30.26 (0.11)	< 0.0001
Ethnicity [*n* (%)]			< 0.0001
Non‐Hispanic White	3822 (78.58)	2821 (75.12)	
Non‐Hispanic Black	1465 (8.71)	1737 (12.51)	
Hispanic	1410 (6.91)	1369 (8.04)	
Other races	550 (5.80)	291 (4.33)	
Educational level [*n* (%)]			0.001
< 9th grade	1253 (9.36)	1151 (10.45)	
9th–11th grade	1020 (11.46)	1030 (13.38)	
High school graduate	1799 (27.66)	1547 (28.90)	
College	1785 (28.00)	1554 (27.12)	
College graduate	1370 (23.52)	919 (20.15)	
PIR [mean (SEM)]	2.93 (0.04)	2.82 (0.04)	0.002
Smoking status [*n* (%)]			< 0.0001
Never	3636 (49.30)	2936 (48.14)	
Former	2874 (41.94)	2417 (39.71)	
Current	727 (8.76)	859 (12.15)	
Frailty index score [mean (SEM)]	0.20 (0.00)	0.21 (0.00)	< 0.001
Frailty status [*n* (%)]			< 0.001
No	5030 (73.17)	4067 (69.48)	
Yes	2217 (26.83)	2151 (30.52)	
Physical activity, MET [mean (SEM)]	2376.29 (79.06)	2675.76 (111.21)	0.01
Physical activity‐Tertiles [*n* (%)]			0.03
Tertile‐1	1538 (33.71)	1254 (33.30)	
Tertile‐2	1762 (40.61)	1353 (37.95)	
Tertile‐3	1079 (25.68)	1012 (28.75)	
COPD			0.48
No	5837 (91.32)	4974 (90.87)	
Yes	506 (8.68)	431 (9.13)	
Hyperlipidemia			0.33
No	1216 (15.24)	1067 (14.46)	
Yes	6029 (84.76)	5149 (85.54)	
DM			0.25
No	4640 (69.05)	3940 (67.63)	
Yes	2607 (30.95)	2278 (32.37)	
Anemia			0.38
No	5956 (88.85)	5011 (88.25)	
Mild	792 (9.02)	759 (9.13)	
Moderate	188 (2.09)	211 (2.55)	
Severe	6 (0.04)	7 (0.07)	

Abbreviations: BMI, body mass index; COPD, chronic obstructive pulmonary disease; DM, diabetes mellitus; MET, metabolic equivalent tasks; PIR, poverty–income ratio; SEM, standard error; SSB, sugar‐sweetened beverage.

**TABLE 2:** The result of univariate analyses using logistic regression model.

Variables	OR (95% CI)	*p*
Age, years	1.04 (1.04, 1.05)	< 0.0001
Sex		
Male	Ref	Ref
Female	1.26 (1.15, 1.38)	< 0.0001
BMI, kg/m^2^	1.05 (1.05, 1.06)	< 0.0001
Ethnicity		
Non‐Hispanic White	Ref	Ref
Non‐Hispanic Black	1.44 (1.29, 1.61)	< 0.0001
Hispanic	1.40 (1.21, 1.63)	< 0.0001
Other races	1.33 (1.02, 1.72)	0.03
Educational level		
< 9th grade	Ref	Ref
9th–11th grade	0.78 (0.66, 0.92)	0.003
High school graduate	0.57 (0.48, 0.66)	< 0.0001
College	0.52 (0.44, 0.60)	< 0.0001
College graduate	0.31 (0.26, 0.38)	< 0.0001
PIR	0.71 (0.69, 0.74)	< 0.0001
Smoking status		
Never	Ref	Ref
Former	1.21 (1.09, 1.34)	< 0.001
Current	1.50 (1.28, 1.77)	< 0.0001
Physical activity, MET	1.00 (1.00, 1.00)	0.1
Physical activity‐Tertiles		
Tertile‐1	Ref	Ref
Tertile‐2	0.80 (0.66, 0.96)	0.02
Tertile‐3	0.69 (0.57, 0.84)	< 0.001
SSB, kcal	1.00 (1.00, 1.00)	0.001
SSB		
No	Ref	Ref
Yes	1.20 (1.09, 1.32)	< 0.001

Abbreviations: BMI, body mass index; MET, metabolic equivalents; OR, odds ratio; PIR, poverty‐income ratio; SSB, sugar‐sweetened beverage.

**TABLE 3:** The results of multivariate analyses using the logistic regression model.

Variables	Model I		Model II		Model III	
	OR (95% CI)	*p*	OR (95% CI)	*p*	OR (95% CI)	*p*
SSB						
No	Ref		Ref		Ref	
Yes	1.20 (1.09, 1.32)	< 0.001	1.25 (1.13, 1.39)	< 0.0001	1.18 (1.02, 1.37)	0.02

*Note:* Model I did not adjust for other covariates. Model II adjusted for age, sex, and BMI. Model III adjusted for Model II plus PIR, physical activity tertiles, educational level, ethnicity, and smoking status.

## 3 Results

### 3.1 Characteristics of Participants

The study included a total of 13,465 participants, with 7247 identified as non‐consumers of SSBs and 6218 as consumers. The mean age of non‐consumers was 71.26 years, which was significantly higher than the mean age of consumers at 69.97 years (*p* < 0.0001). In terms of gender distribution, a higher proportion of males were found in the consumer group compared to the non‐consumer group (*p* = 0.003). BMI analysis revealed that non‐consumers had a lower mean BMI of 29.41 kg/m^2^, while consumers had a mean BMI of 30.26 kg/m^2^ (*p* < 0.0001). Ethnic composition showed that non‐Hispanic Whites were the majority in both groups. However, a greater percentage of SSB consumers were non‐Hispanic Blacks (12.51%) and Hispanics (8.04%) compared to non‐consumers (8.71% and 6.91%, respectively) (*p* < 0.0001). Educational attainment differed significantly between the groups (*p* = 0.001), with a higher percentage of non‐consumers (51.52%) achieving higher education levels than consumers (47.27%). The mean PIR was slightly higher for non‐consumers (2.93) than consumers (2.82) (*p* = 0.002). Smoking status indicated that a larger percentage of SSB consumers were current smokers (12.15%) compared to non‐consumers (8.76%) (*p* < 0.0001). The prevalence of chronic conditions was also assessed: chronic obstructive pulmonary disease (COPD) was present in 9.45% of SSB consumers versus 7.82% in non‐consumers (*p* = 0.02); diabetes mellitus (DM) was reported by 14.32% of consumers compared to 11.67% of non‐consumers (*p* = 0.01); hyperlipidemia affected 22.58% of consumers and 18.92% of non‐consumers (*p* = 0.03); and anemia was more prevalent among SSB consumers at 15.63% versus 12.78% in non‐consumers (*p* = 0.04). The mean frailty index score was higher among consumers (0.21) than among non‐consumers (0.20) (*p* < 0.001), with a greater proportion of consumers (30.52%) classified as frail compared to non‐consumers (26.83%) (*p* < 0.001). Finally, physical activity levels were assessed, showing that consumers had a higher mean level of physical activity measured in MET at 2675.76 compared to non‐consumers at 2376.29 METs (*p* = 0.01). Table 1 shows the characteristics of participants.

### 3.2 The Correlation Between Frailty and Covariates

In the univariate logistic regression analysis, several covariates were significantly associated with frailty among older adults with hypertension (Table 2). Age and BMI were positively correlated with frailty. Compared to males, females had higher odds of being frail. Ethnicity was also associated with frailty, with non‐Hispanic Blacks, Hispanics, and other races having higher odds of frailty compared to non‐Hispanic Whites. Higher educational levels and higher PIR were associated with lower odds of frailty. Smoking status was significantly correlated with frailty, as former smokers and current smokers had higher odds of being frail compared to never‐smokers. Physical activity, categorized into tertiles, showed a protective effect against frailty, with the second and third tertiles having lower odds of frailty compared to the lowest tertile. Lastly, SSB consumption was positively associated with frailty, as individuals who consumed SSBs had higher odds of being frail than those who did not.

### 3.3 The Relationship Between SSB and Frailty in Elderly Hypertensive Individuals

The multivariate logistic regression analysis revealed a significant association between SSB consumption and frailty in older adults with hypertension (Table 3). In Model I, which did not adjust for any covariates, the odds ratio (OR) for frailty among SSB consumers was 1.20 (95% confidence interval [CI]: 1.09–1.32, *p* < 0.001) compared to non‐consumers. After adjusting for age, sex, and BMI in Model II, the OR increased to 1.25 (95% CI: 1.13–1.39, *p* < 0.0001). Further adjustments in Model III, which included additional potential confounders such as PIR, physical activity, educational level, ethnicity, and smoking status, maintained a statistically significant association with an OR of 1.18 (95% CI: 1.02–1.37, *p* = 0.02). These results suggested that SSB consumption was independently associated with higher levels of frailty among older adults with hypertension, regardless of demographic, socioeconomic, and lifestyle characteristics.

**FIGURE 2:**
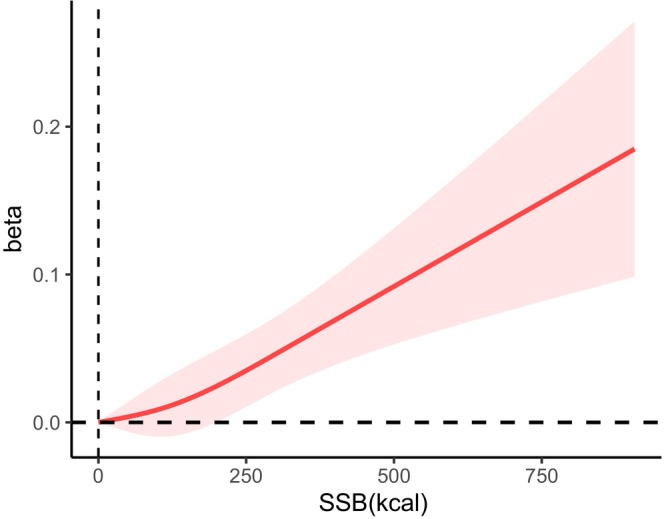
Curvilinear relationship between SSB consumption and frailty risk. A curvilinear relationship between SSB consumption and frailty risk is close to linear after adjusting for age, sex, BMI, PIR, physical activity tertiles, educational level, ethnicity, and smoking status. Beta is the effect value of the frailty index difference.

Additionally, the analysis explored the relationship between SSB consumption and the frailty index score as continuous variables through restricted cubic spline curve fitting. The findings revealed a nearly linear relationship between SSB intake and frailty index scores after adjusting for relevant covariates (Figure 2).

## 4 Discussion

According to our analysis, SSB consumption was positively related to frailty among older adults with hypertension. Even after adjusting for variables such as age, sex, BMI, PIR, physical activity level, educational attainment, ethnic background, and smoking status, this correlation remained statistically significant. In addition, the relationship exhibited a linear trend between total SSB consumption and frailty.

These findings are consistent with previous research that has linked SSB consumption to various adverse health outcomes, including obesity, type 2 diabetes, and cardiovascular disease (Malik et al. 2010; Pietrantoni and Mayrovitz 2022). A meta‐analysis found an association between SSB consumption and metabolic syndrome and type 2 diabetes (Malik et al. 2010). Additionally, Struijk et al. identified a higher risk of frailty associated with SSB consumption in a cohort of older women (Struijk et al. 2020). Laclaustra et al. further corroborated that added sugar consumption correlates with frailty in older adults (Laclaustra et al. 2018). However, our study uniquely focuses on the specific association between SSB intake and frailty in older adults suffering from hypertension.

The mechanisms underlying the association between SSB consumption and frailty are not fully understood. However, several hypotheses have been proposed. One possibility is that SSB consumption may lead to increased inflammation, oxidative stress, and insulin resistance (Ma et al. 2022), factors known to influence frailty. Alternatively, SSB may displace nutrient‐dense foods in the diet, leading to malnutrition and muscle wasting (Malik and Hu 2022).

Our study has several strengths. It addresses a gap in the literature by focusing on dietary impacts on frailty specifically in hypertensive patients. Our analysis utilized logistic regression and restricted cubic splines to clarify the relationship between SSB consumption and frailty. Furthermore, we examined this relationship using both continuous and categorical variables.

Nevertheless, various constraints in our research need to be taken into account. The study's focus on elderly individuals with hypertension may limit the generalizability of our findings to other populations. Additionally, while we accounted for several confounding variables, unmeasured confounding could still affect our results due to unidentified factors. Moreover, the cross‐sectional design of the study prevents us from establishing causal relationships.

In conclusion, this study provides evidence for a positive association between SSB consumption and frailty in old adults with hypertension. Additional investigation is required to validate our findings and clarify the underlying processes.

## Author Contributions

**Si Cao:** formal analysis (equal), writing – review and editing (equal). **Youjie Zeng:** writing – original draft (equal). **Sai Zhou:** formal analysis (equal). **Wenming Song:** writing – review and editing (equal). **Gong Chen:** project administration (equal), writing – review and editing (equal).

## Ethics Statement

NCHS’ Research Ethics Review Board approved the studies involving humans. Studies were conducted in accordance with local laws and institutional requirements. Informed consent was obtained from the participants.

## Conflicts of Interest

The authors declare no conflicts of interest.

## Data Availability

All the data are available to the public and were used in the manuscript. The data can be available on the website: https://wwwn.cdc.gov/nchs/nhanes/search/default.aspx.
